# Hemophagocytic Syndrome-Like Tuberculosis-Immune Reconstitution Inflammatory Syndrome After the Initiation of Hepatic Tuberculosis Treatment

**DOI:** 10.7759/cureus.24644

**Published:** 2022-05-01

**Authors:** Serina Nakamura, Naokazu Muramae, Akira Fujisawa, Noriaki Yasuda, Mitsumasa Okano, Kenta Mori, Kazunori Otsui, Kazuhiko Sakaguchi

**Affiliations:** 1 Internal Medicine, Center for Medical Education and Training, Kobe University Hospital, Kobe, JPN; 2 General Internal Medicine, Kobe University Hospital, Kobe, JPN

**Keywords:** steroid, hemophagocytic syndrome, tb-iris, initial deterioration, hepatic tuberculosis

## Abstract

A 25-year-old Nepalese woman was referred to our hospital because of fever and intermittent abdominal pain with inguinal lymphadenopathy, which had lasted for several months. A liver biopsy of the positron emission tomography-positive lesion led to a diagnosis of hepatic tuberculosis. After the initiation of antituberculosis treatment, her symptoms resolved. However, 11 days after treatment initiation, chest and back pain, high-grade fever, and vomiting appeared and gradually worsened. She developed anemia and her serum ferritin level was elevated. Hemophagocytic syndrome due to the initial deterioration of tuberculosis was suspected and steroid therapy was initiated with the continuation of the antituberculosis drugs. Thereafter, the patient’s condition improved remarkably.

## Introduction

Tuberculosis (TB) remains a significant global health concern. Asia is an endemic region for TB, and in Nepal, it is estimated that approximately 45% of the total population is infected with TB, with 20,000 new infections reported each year [1].

TB, particularly extrapulmonary TB (EPTB), is an important cause of fever of unknown origin. Among EPTB, hepatic TB is rare, and its symptoms are nonspecific, including fever, weight loss, and malaise. However, unexpected hepatomegaly may serve as a diagnostic cue. Because imaging studies alone are not sufficient, a definitive diagnosis is made based on histological and bacterial findings in a liver biopsy sample [2,3]. Early intervention with antituberculosis medication can lead to a good prognosis [4]. However, the patient's condition occasionally deteriorates after initiating antituberculosis medication [5], which is called initial deterioration of tuberculosis-immune reconstitution inflammatory syndrome (TB-IRIS). This condition is likely to occur in patients with EPTB because of a high bacterial load.

Herein, we report a case of hepatic TB that presented with a hemophagocytic syndrome-like manifestation due to initial deterioration of TB-IRIS.

## Case presentation

**Figure 1:**
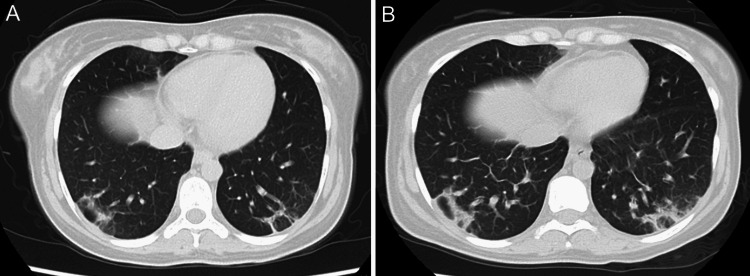
Plain chest CT scan image (A) At the first hospitalization, slight reticular shadows in the bilateral dorsal lower lung field were seen. (B) At the time of the second hospitalization, the reticular shadows slightly increased.

**Figure 2:**
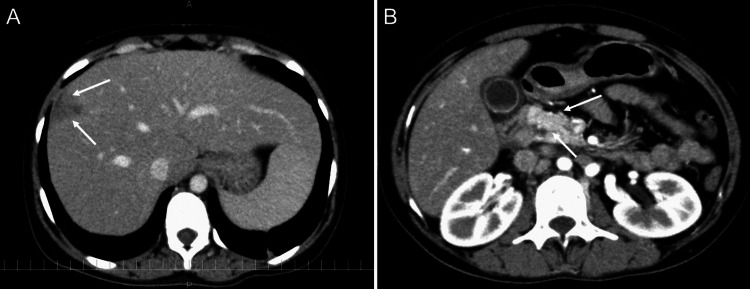
Abdominal enhanced CT scan image at the first hospitalization (A) Irregular mass shadows in the S8 region of the liver (white arrows) and (B) para-aortic lymphadenopathy (white arrows) were seen.

**Figure 3:**
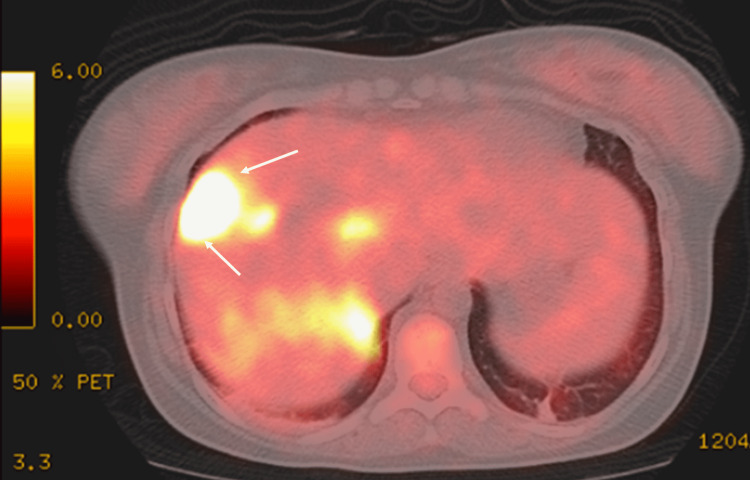
FDG-PET/CT image at the first hospitalization Abnormal accumulation in the S8 region of the liver (white arrows) is shown. FDG-PET/CT: 18F-fluorodeoxyglucose positron emission tomography/computed tomography.

A 25-year-old Nepalese woman was admitted to our hospital with high-grade fever and intermittent abdominal pain with inguinal lymphadenopathy, which had lasted for two months. She had lived in Japan for one year. Chest and abdominal computed tomography (CT) scans obtained at a previous clinic showed generalized lymphadenopathy with calcification. A biopsy of the inguinal lymph nodes was performed, with no significant findings. At the time of admission to our hospital, plain chest CT showed slight reticular shadows in the bilateral dorsal lower lung field (Figure 1), and abdominal contrast-enhanced CT showed irregular mass shadows in the S8 region of the liver (Figure 2A), with para-aortic lymphadenopathy (Figure 2B). 18F-fluorodeoxyglucose positron emission tomography/computed tomography (FDG-PET/CT) revealed abnormal accumulation in the S8 region of the liver (Figure 3).

**Figure 4:**
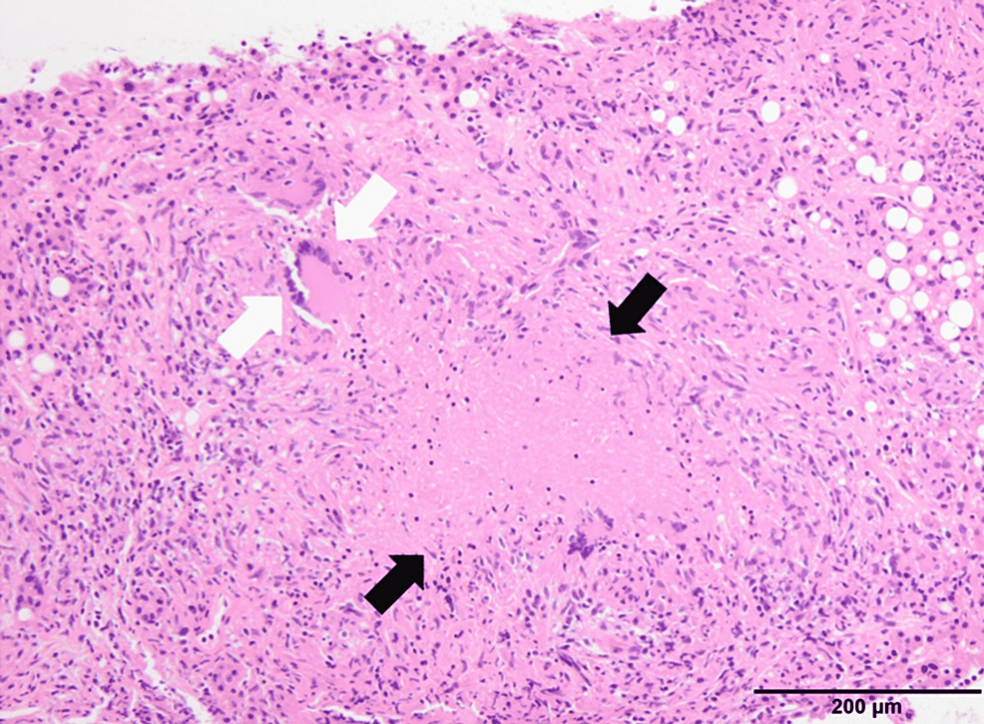
Hematoxylin and eosin staining of liver biopsy sample (10×) Multiple granulomas with necrosis (black arrows) and multinucleated giant cell aggregation (white arrows) indicated necrotic epithelial granulomas.

After performing antibody tests, HIV, cytomegalovirus (CMV), and Epstein-Barr virus (EBV) infections were excluded as the causes of unknown fever. There were no findings strongly suggestive of malignancy or autoimmune disease. TB was suspected because of its high incidence in Nepal, and the TB-specific interferon-gamma release assay (T-SPOT) was positive. A liver biopsy was performed. Pathological findings included multiple granulomas with necrosis, multinucleated giant cell clusters, and inflammatory cell infiltrates such as lymphocytes, plasma cells, and eosinophils in the portal region (Figure 4).

This necrotizing epithelioid granuloma finding was compatible with a mycobacterial infection such as TB. Culture tests revealed *Mycobacterium tuberculosis*, leading to a definitive diagnosis of hepatic TB. Treatment with antituberculosis drugs, i.e., isoniazid (INH) 250 mg, rifampicin (RFP) 300 mg, ethambutol (EB) 750 mg, and pyrazinamide (PZA) 1000 mg, was initiated. Her general condition improved remarkably with the resolution of high-grade fever, and she was discharged to continue antituberculosis medications for six months. However, five days after discharge (nine days after initiating antituberculosis treatment), she visited the emergency room and was readmitted because of fever and abdominal pain that started three days before.

On physical examination, the patient appeared to be ill. Her body temperature was 38.2°C, her blood pressure was 101/56 mmHg, pulse was 120 beats per minute, respiratory rate was 24 breaths per minute, and oxygen saturation was 100% while breathing ambient air. Her conjunctiva was pale, but not icteric, and she had right cervical lymphadenopathy with pain. In addition, she experienced spontaneous pain and tenderness from the epigastrium to the right hypochondrium. Cardiac and pulmonary examination findings were normal. The laboratory data on admission are presented in Table 1.

**Table 1:** Laboratory data on admission WBC: white blood cell count; Stab: stab-shaped neutrophil; Seg: segmented neutrophil; Eosi: eosinophil; Mono: monocyte; Lymph: lymphocyte; RBC: red blood cell count; Hb: hemoglobin; Plt: platelet count; APTT: activated partial thromboplastin time; PT-INR: prothrombin time-international normalized ratio; Fib: fibrinogen; TP: total protein; Alb: albumin; T-bil: total bilirubin; AST: aspartate aminotransferase; ALT: alanine transaminase; γ-GTP: γ-glutamyl transferase; LDH: lactate dehydrogenase; BUN: blood urea nitrogen; Cre: creatinine; Na: sodium; K: potassium; Cl: chloride; cCa: corrected calcium; TG: triglyceride; Glu: glucose; CRP: C-reactive protein.

Test	Result	Units	Reference range
WBC	3700	/μL	3300-8600
Stab	10	%	2-13
Seg	78	%	38-58
Eosi	1	%	0-5
Mono	2	%	2-8
Lymph	9	%	26-47
RBC	277	×10^4^/μL	386-492
Hb	7.5	g/dL	11.6-14.8
Plt	14.8	×10^4^/μL	15.8-34.8
APTT	52.9	sec	26-38
PT-INR	1.34		0.85-1.15
Fib	182	mg/dL	200-400
TP	5.9	mg/dL	6.6-8.1
Alb	2.5	mg/dL	4.1-5.1
AST	213	IU/L	13-30
ALT	79	IU/L	7-23
γ-GTP	207	IU/L	9-32
LDH	703	IU/L	124-222
T-bil	1.1	mg/dL	0.4-1.5
BUN	5.2	mg/dL	8-20
Cre	0.43	mg/dL	0.46-0.79
Na	123	mEq/L	138-145
K	3.1	mEq/L	3.6-4.8
Cl	94	mEq/L	101-108
cCa	9.3	mg/dL	8.8-10.1
TG	169	mg/dL	28-149
Glu	126	mg/dL	73-109
Ferritin	1815	ng/mL	5-152
CRP	6.92	mg/dL	0.8-1.0

The WBC count was 37,000 per cubic millimeter, hemoglobin (Hb) level was 7.5 g per deciliter, and platelet count was 148,000 per cubic millimeter. The levels of C-reactive protein (CRP), ferritin, and liver-leaking enzymes were elevated. Plain CT showed slightly increased reticular shadows in the bilateral dorsal lower lung field (Figure 1B). The patient’s ferritin level reached 5,900 ng/mL on the second day of hospitalization. Hemophagocytic syndrome was suspected because of high-grade fever, anemia, and liver dysfunction. No other basal conditions for the hemophagocytic syndrome, such as viral infection or malignancy, were found. Treatment failure of TB due to drug-resistant *Mycobacterium* was considered a differential diagnosis, but due to the low rate of drug-resistant *M. tuberculosis* in Nepal [6], this possibility was eventually ruled out. In fact, the sensitivity of antituberculosis medication, which was used in this case, was high (Table 2).

**Table 2:** Drug susceptibility of antituberculosis medication CPFX: ciprofloxacin; LVFX: levofloxacin; SM: streptomycin; INH: isoniazid; KM: kanamycin; EB: ethambutol; RFP: rifampicin; RBT: rifabutin; PZA: pyrazinamide.

Medication	Susceptibility (MIC, μg/mL)
CPFX	Sensitive (0.25)
LVFX	Sensitive (0.25)
SM	Sensitive (0.5)
INH	Sensitive (0.125)
KM	Sensitive (1)
EB	Sensitive (1)
RFP	Sensitive (<0.03)
RBT	Sensitive (0.008)
PZA	Sensitive (1)

Since her condition developed after the initiation of the antituberculosis medication, the initial deterioration caused by antituberculosis treatment was deemed to be the main cause of her symptoms. In addition, hemophagocytic syndrome due to immune reconstitution associated with TB treatment has been speculated [7]. Bone marrow aspiration biopsy was not performed as she refused consent. However, since her condition deteriorated, we decided to initiate steroid therapy with the continuation of the antituberculosis medication. Steroid pulse therapy (intravenous methylprednisolone 1,000 mg/day) was administered for three days starting on the second day of hospitalization, followed by a maintenance dose of oral prednisolone (40 mg/day).

**Figure 5:**
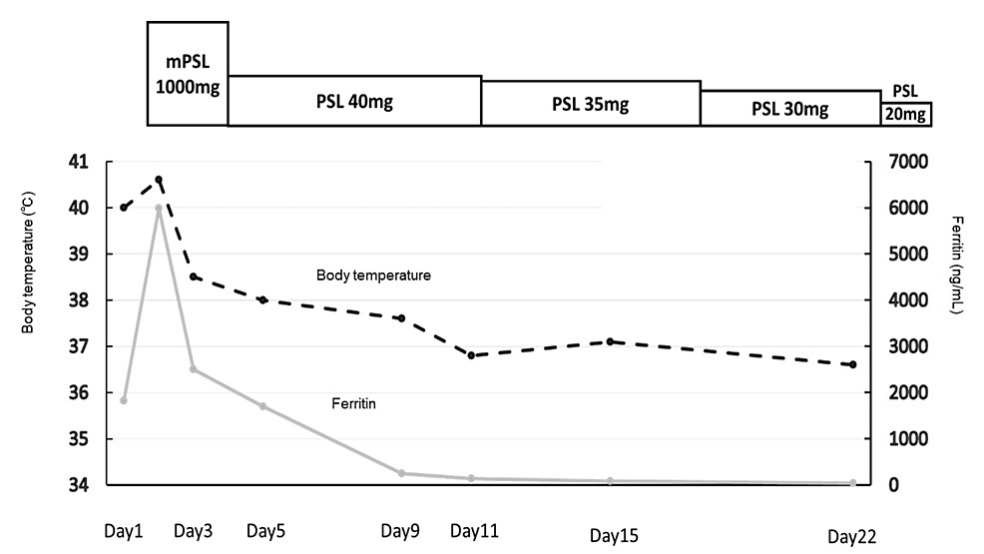
The clinical course at the second hospitalization mPSL: methylprednisolone; PSL: prednisolone.

By the ninth day of hospitalization, she was afebrile, and her anemia and ferritin levels had normalized. The patient was discharged on the 22nd day of hospitalization with prednisolone (20 mg/day); the dose was intended to taper gradually at the outpatient clinic. Figure 5 shows the changes in steroid dosage, serum ferritin levels, and serum CRP levels.

## Discussion

The manifestations of TB-IRIS include the development of new lesions, worsening of existing lesions, recurrence of fever, lymphadenopathy, and dyspnea during or after the completion of antituberculosis therapy. It is essential to rule out other causes such as the emergence of resistant mycobacteria, poor drug absorption, and poor compliance to avoid misinterpreting the results as treatment failure [5]. TB-IRIS is considered an excessive immune response to *M. tuberculosis*, which occurs especially in EPTB, where the number of bacteria is higher compared with that in pulmonary TB [8,9]. It is reported that 82.8% of patients with TB-IRIS have EPTB [5]. TB-IRIS is known to occur in HIV patients [10], but there have been reports of TB-IRIS in non-HIV patients also, and its occurrence should be considered regardless of HIV infection [11].

TB-IRIS occurs in various organs, including the central nervous system, respiratory system, skin, soft tissues, lymph nodes, bones, and abdomen, with the respiratory system being the most frequently affected. The median time from the initiation of antituberculosis drugs to TB-IRIS development is reported to be 60 days [11]. In the present case, fever and abdominal pain developed on the 10th day after the initiation of treatment.

Although the standard of care for TB-IRIS is not clearly defined, most cases improve with the continuation of antituberculosis medication [5]. Surgical procedures, such as abscess drainage and steroid administration, if necessary, can shorten the duration of treatment [8,9]. It is essential to exclude the emergence of drug-resistant mycobacteria before starting prednisolone [11,12].

Hemophagocytic syndrome is a lethal inflammatory disease. Although it can occur in any age group, 40% of the hemophagocytic syndrome has been reported to occur in adults [13]. Hemophagocytic syndrome in adults is classified as primary (idiopathic) or secondary, and secondary causes include infections (viral, bacterial, parasitic, and fungal), malignancy, and autoimmunity. Hemophagocytic syndrome triggered by bacterial infections accounts for 9% of cases, of which 38% are caused by *M. tuberculosis* [13]. Thus, TB itself also triggers hemophagocytic syndrome, and the mortality rate is estimated to be 50% [7]. In contrast, there are a few reports of hemophagocytic syndrome emergence after initiating antituberculosis medications, as in the case of our patient [14]. T cell-mediated immunity is thought to be involved in pathogenesis, affecting cytokines such as interferon gamma (IFN-γ), interleukin (IL)-2, and IL-6, which cause hemophagocytic syndrome [14,15]. Suppression of the cytokine storm is vital for treating hemophagocytic syndrome, and steroid pulse therapy was effective in this case.

## Conclusions

In conclusion, in the treatment of TB, especially in extrapulmonary cases, clinicians should be aware that hemophagocytic syndrome may occur due to the initial deterioration of TB-IRIS, and unlike hemophagocytic syndrome caused by TB itself, the prognosis may be better with early and appropriate treatment.
